# Efficacy and tolerability of the combination of minocycline and metronidazole for macrolide-resistant *Mycoplasma genitalium*

**DOI:** 10.1093/jac/dkaf142

**Published:** 2025-05-22

**Authors:** Kay Htaik, Lenka A Vodstrcil, Erica L Plummer, Laura G Matthews, Ivette Aguirre, Eric P F Chow, Christopher K Fairley, Catriona S Bradshaw

**Affiliations:** Melbourne Sexual Health Centre, Alfred Health, Melbourne, Victoria, Australia; School of Translational Medicine, Monash University, Melbourne, Victoria, Australia; Melbourne Sexual Health Centre, Alfred Health, Melbourne, Victoria, Australia; School of Translational Medicine, Monash University, Melbourne, Victoria, Australia; Centre for Epidemiology and Biostatistics, Melbourne School of Population and Global Health, The University of Melbourne, Melbourne, Victoria, Australia; Melbourne Sexual Health Centre, Alfred Health, Melbourne, Victoria, Australia; School of Translational Medicine, Monash University, Melbourne, Victoria, Australia; Melbourne Sexual Health Centre, Alfred Health, Melbourne, Victoria, Australia; School of Translational Medicine, Monash University, Melbourne, Victoria, Australia; Melbourne Sexual Health Centre, Alfred Health, Melbourne, Victoria, Australia; Melbourne Sexual Health Centre, Alfred Health, Melbourne, Victoria, Australia; School of Translational Medicine, Monash University, Melbourne, Victoria, Australia; Centre for Epidemiology and Biostatistics, Melbourne School of Population and Global Health, The University of Melbourne, Melbourne, Victoria, Australia; Melbourne Sexual Health Centre, Alfred Health, Melbourne, Victoria, Australia; School of Translational Medicine, Monash University, Melbourne, Victoria, Australia; Melbourne Sexual Health Centre, Alfred Health, Melbourne, Victoria, Australia; School of Translational Medicine, Monash University, Melbourne, Victoria, Australia; Centre for Epidemiology and Biostatistics, Melbourne School of Population and Global Health, The University of Melbourne, Melbourne, Victoria, Australia

## Abstract

**Background and objectives:**

Curing *Mycoplasma genitalium* is challenging in the context of rising antimicrobial resistance and limited therapeutic options. There is an urgent need for globally relevant and effective treatment options using readily available and affordable agents. From September 2021 to August 2024 at Melbourne Sexual Health Centre, we prospectively evaluated microbial cure and tolerability of oral minocycline 100 mg combined with metronidazole 400 mg (twice daily for 14 days) for individuals with macrolide-resistant *M. genitalium* in whom fluroquinolones had failed or were not advised.

**Methods:**

Microbial cure was defined as a negative test of cure (TOC) using transcription-mediated amplification 14–90 days after completing the regimen. The proportion cured and 95% confidence intervals (CIs) were calculated. Data on side effects and adherence were collected at TOC visits.

**Results:**

Microbial cure in patients receiving the combination regimen was 80.8% (95% CI: 71.9–87.8%). Cure in those who had received preceding doxycycline was 90.3% (*n* = 28/31, 95% CI: 74.2–98.0%) compared to 76.7% (*n* = 56/73, 95% CI: 65.4–85.8%) in those who had not, *P* = 0.172. Central nervous system and gastrointestinal side effects were commonly reported.

**Conclusions:**

Minocycline with metronidazole cured 80% of macrolide-resistant infections in this cohort. Cure may be enhanced by the use of doxycycline before the combination regimen but larger studies are needed. Given limited options for treating resistant *M. genitalium* infections, the combined minocycline and metronidazole regimen may represent a promising option where no alternative drugs are available, or quinolones are contraindicated. Clinicians should be aware of and discuss side effects with patients.

## Introduction

Curing *Mycoplasma genitalium* is challenging due to rising antimicrobial resistance and limited therapeutic options. The prevalence of 23S rRNA gene mutations conferring resistance to macrolides has increased globally and continues to rise.^1,2^ In Australia, there has been an increase in macrolide-resistant *M. genitalium* from 19% in 2010 to 66% by 2017, which is particularly notable among men who have sex with men, where resistance exceeds 80%.^1^ Moxifloxacin is the first-line antimicrobial recommended for macrolide-resistant infections, but mutations in the *parC* region, which have been associated with resistance to fluoroquinolones, are also rising.^2^ The most common *parC* amino acid change, S83I, is associated with failure of moxifloxacin in ∼60% of infections.^3^ Alternate antimicrobials are urgently needed. Options have included minocycline, pristinamycin or sitafloxacin.^4–7^

Pristinamycin cures 75% (95% CI: 66–82%) of macrolide-resistant *M. genitalium* infections but is increasingly difficult to access as it is manufactured by only one company in France and is subject to export restrictions which limit its access outside Europe.^4,7^ Sitafloxacin, an extended spectrum fluoroquinolone with a higher efficacy *in vitro* and *in vivo* than moxifloxacin for *M. genitalium* is also difficult to access in many countries, and has recently also been subject to export restrictions.^6,8^ Furthermore, in Australia, in the interest of antimicrobial stewardship and resistance, it has generally been recommended that sitafloxacin be reserved for the treatment of serious infections where possible.

Minocycline is available as a generic agent and has been shown to cure ∼68% (95% CI: 58–76%) of macrolide-resistant *M. genitalium* infections when used in a 14-day regimen.^5^ This is higher than the reported proportion cured following 14 days of doxycycline monotherapy (58.9%, 95% CI: 52.7–64.9%) and is supported by *in vitro* data that shows *M. genitalium* is more susceptible to minocycline than doxycycline.^8,9^ Importantly, a randomized controlled trial unexpectedly found that the addition of metronidazole, to doxycycline and ceftriaxone for the treatment of acute pelvic inflammatory disease modestly improved the cure of *M. genitalium* compared to women treated with only ceftriaxone and doxycycline.^10^ This finding was subsequently supported by *in vitro* data that showed metronidazole has activity against *M. genitalium*.^11^ We hypothesized that the addition of metronidazole to minocycline may be more effective for the treatment of *M. genitalium* infections than minocycline alone. In the current study, we evaluated the efficacy and tolerability of 14 days of minocycline with metronidazole (the combination regimen) for the treatment of *M. genitalium* infections amongst patients attending the Melbourne Sexual Health Centre (MSHC), the largest publicly funded sexual health service in Australia with >50 000 consultations per year.

## Methods

This was a prospective study of patients prescribed a combination of minocycline and metronidazole for macrolide-resistant *M. genitalium* infections at MSHC between September 2021 and August 2024. Ethics approval for this study was provided by the Alfred Hospital Ethics Committee (approval number 232/16).

Patients were eligible for inclusion if they had a urogenital or rectal *M. genitalium* infection, were prescribed a combination of oral minocycline 100 mg twice daily and oral metronidazole 400 mg twice daily for 14 days, completed at least 50% of both antibiotic courses (i.e. at least 7 days or 14 doses of each drug) and returned for a test of cure (TOC) 14–90 days after completing the combination regimen. Patients with a high risk of reinfection were excluded; this was defined as condomless penetrative vaginal and/or anal intercourse with an untested and/or untreated or partially treated ongoing partner since diagnosed with *M. genitalium* infection, in keeping with previous studies.^5,6,7,12^

At MSHC, it is a routine practice for *M. genitalium* positive patients to be recalled for a TOC 14–28 days after completing treatment. During this visit, clinicians document resolution of symptoms, adherence, side effects, sexual activity, condom use and treatment status of sex partner(s) in a standardized electronic TOC template.

During the study period, all samples (before treatment and at TOC) were tested for *M. genitalium* using the *M. genitalium* transcription-mediated amplification (TMA) assay (Aptima Hologic Gen-Probe Panther System; Hologic, San Diego, CA, USA). Samples testing positive for *M. genitalium* by TMA were then tested using the ResistancePlus MG assay (SpeeDx Pty Ltd, Sydney, Australia) to determine the macrolide-resistance result to inform resistance-guided therapy.^13,14^ During the study period, clinicians prescribed the combination regimen for patients with macrolide-resistance mutations detected, for which fluoroquinolones had failed or were contraindicated or not tolerated. Microbial cure was defined as a negative TOC within 14–90 days of completing treatment. The proportion with microbial cure and 95% confidence intervals (CIs) were calculated by exact methods. Univariable logistic regression was used to explore characteristics associated with treatment failure. All statistical analyses were conducted in STATA v.17 (StataCorp LP, College Station, TX, USA).

## Results

**Figure 1.**
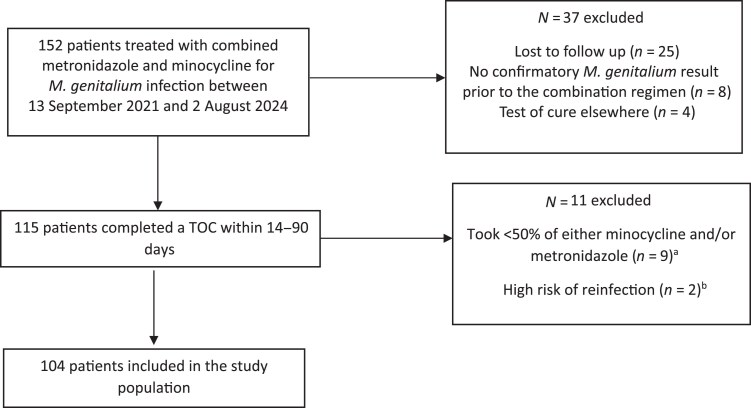
Study selection. ^a^Treatment limiting side effects experienced by nine patients who took <50% of either of the antibiotics include the following: Patient 1: numb tongue, metallic tase, dry mouth; Patient 2: headache, fatigue, dizziness; Patient 3: palpitation, disorientation, unsteady gait; Patient 4: unsteady gait; Patient 5: poor judgement, confusion; Patient 6: depersonalization; Patient 7: fatigue and dizziness; Patient 8: flicking eyelid, feeling spaced out, reduced concentration; Patient 9: missed all doses. Unknown reason. ^b^Reported condomless penetrative intercourse with an untreated/partially treated ongoing partner since being diagnosed with *M. genitalium* infection.

During the study period, 152 patients with an *M. genitalium* infection were treated with combination minocycline and metronidazole. Forty-eight patients were excluded from the analysis because: they did not return for a TOC within 14–90 days of treatment (*n* = 25), had no confirmatory *M. genitalium* result at MSHC before commencing the combination regimen (*n* = 8), completed a TOC at a service other than MSHC (*n* = 4), took <50% of their course of minocycline and/or metronidazole (*n* = 9) or were classified as at high risk of reinfection (*n* = 2). A total of 104 patients were included in the current analysis (Figure 1).

### Population characteristics

The median age of the study population was 32 years (IQR = 20 to 54); 34 (33%) were women, 22 (21%) were men reporting female partners only and 47 (45%) were men who were gay, bisexual or other men who have sex with men (GBMSM) (Table 1). Three (3%) individuals were known to be living with HIV (human immunodeficiency virus) and 32 (31%) reported current use of pre-exposure prophylaxis (PrEP) for HIV. Symptoms were reported by 58 (56%) individuals at the time of initial presentation for the combination regimen. Most (90, 87%) individuals eligible for inclusion had macrolide-resistant *M. genitalium* confirmed by testing at MSHC. The remaining 14 (13%) of patients had resistance results that were unknown or unavailable and received the combination regimen as they had failed one or more previous regimens and were considered to have macrolide-resistant infection.

**Table 1.** Characteristics of the study population

	Total (*n* = 104) *n* (%)
Age in years, median (range)	32 (20–54)
Sex and sexual orientation	
Female	34 (32.7)
Men reporting female partners only	22 (21.2)
Gay, bisexual and other men who have sex with men	47 (45.2)
Transgender male^a^	1 (1.0)
HIV status	
Living with HIV	3 (2.9)
HIV negative or not known to be HIV positive	101 (97.1)
PrEP use	
Yes	32 (30.8)
No	72 (69.2)
Symptom status (at the time of prescription)	
Asymptomatic	46 (44.2)
Symptomatic	58 (55.8)
Indication for testing	
Male urethritis	44 (42.3)
Female pelvic symptoms	8 (7.7)
Female urinary symptoms	4 (3.9)
Rectal symptoms	3 (2.9)
Persistent asymptomatic infection	33 (31.7)
Asymptomatic sexual contact of infection	6 (5.8)
others^b^	6 (5.8)
Site of infection	
First pass urine^c^	64 (62.5)
Cervicovaginal	30 (27.8)
Anorectal	10 (8.6)
Previous antibiotic treatment^d^	
No previous treatment	16 (15.4)
1 previous treatment regimen	39 (37.5)
2+ previous treatment regimens	48 (46.2)
Adherence^e^	
No missed doses documented	98 (94.2)
Missed 1–6 days of either antibiotic	4 (3.9)

^a^One transgender male reported sex with men and women. Their data was included with data from GBMSM for analyses of treatment efficacy.

^b^Two unknown indications, four had persistent change in vaginal discharge after excluding other STIs.

^c^One had multisite infection, both in urine and anorectal sites, only included urine.

^d^One with unknown or missing previous treatment regimen.

^e^Two patients with unknown adherence.

The initial indications for *M. genitalium* testing included male urethritis (*n* = 44, 42%), female pelvic symptoms (*n* = 8, 8%), female urinary symptoms (*n* = 4, 4%), rectal symptoms (*n* = 3, 3%), persistent asymptomatic infection (*n* = 33, 32%) and asymptomatic contact of *M. genitalium* (*n* = 6, 6%). A total of 30 (28%) infections were detected in cervicovaginal samples, 64 (63%) in first pass urine and 10 (9%) in anorectal samples. One male had *M. genitalium* detected in both a urine and an anorectal sample.

Doxycycline 100 mg twice a day for 7 days is used for the treatment of STI syndromes at MSHC (i.e. non-gonococcal urethritis, pelvic inflammatory disease and proctitis) before an *M. genitalium* result becoming available. Thirty-one (30%) patients had received pre-treatment with between 3 and 7 days of doxycycline immediately before commencing the combination regimen and 73 (70%) received the combination regimen without previous doxycycline.

Eighty-seven (84%) patients had received one or more antibiotic regimen/s for *M. genitalium* treatment before receiving the combination regimen. Thirty-nine (38%) had failed one previous regimen, and 48 (46%) had failed two or more previous regimens. Past failed regimens included moxifloxacin (*n* = 68), sitafloxacin (*n* = 38) and minocycline monotherapy (*n* = 11), with small numbers also failing azithromycin, pristinamycin and lefamulin. Sixteen (15%) patients received combination minocycline and metronidazole as their first treatment for *M. genitalium*. The most common reasons for this included the perceived need to (i) avoid quinolones due to pre-existing medical conditions (e.g. tendon injury, heart palpitations) and (ii) avoid potential drug interactions with quinolones (e.g. prolonged QT interval).

### Microbial cure and factors associated with failure

Of the 104 eligible patients who received combination minocycline and metronidazole, 80.8% (95% CI: 71.9–87.8%) experienced microbial cure within 14–90 days of completion of the regimen. The median time from treatment until TOC was 32 days (IQR, 28 to 42 days). Data were then stratified by those who received pre-treatment with doxycycline immediately before combination regimen versus those who did not. Microbial cure in those who received 3–7 days of doxycycline before commencing the combination regimen was 90.3% (*n* = 28/31, 95% CI: 74.2–98.0%) compared to 76.7% (*n* = 56/73, 95% CI: 65.4–85.8%) in those who did not receive doxycycline (*P* = 0.172) (Table 2).

**Table 2.** Influence of doxycycline pre-treatment on efficacy of combined minocycline and metronidazole

	Previous treatment with doxycycline^a^ (*n* = 31) *n*, % (95% CI)	No previous treatment with doxycycline (*n* = 73) *n*, % (95% CI)	*P* value^b^
Cured	28, 90.3 (74.2–98.0)	56, 76.7 (65.4–85.8)	0.172
Failed	3, 9.7 (2.0–25.8)	17, 23.3 (14.2–34.7)	

^a^Patients received 3–7 days of doxycycline before commencing combination therapy with minocycline and metronidazole.

^*b*^ *P* value calculated Fisher’s exact test.

We examined microbial cure by gender and/or sexual orientation, anatomical site of infection, symptomatic status, failure of prievious antimicrobial regimens and adherence (Table 3). A significantly increased risk of microbial failure was observed in men reporting female partners when compared to women (OR: 4.29, 95% CI: 1.10–16.66; *P* = 0.036) (Table 3). Although there were 3-fold increased odds of experiencing treatment failure among those with urethral infections compared to cervicovaginal infections, this was not significant (OR: 3.00, 95% CI: 0.80–11.23; *P* = 0.103). There was also no significant difference in the odds of failing the combination regimen by symptomatic status nor by level of adherence to either regimen, but small numbers affected this comparison (Table 3). As only one factor was associated with treatment failure, multivariable logistic regression analysis was not performed.

**Table 3.** Factors associated with failure

	Cured	Failed		
Factor	*n*, % (95%CI)	*n*, % (95%CI)	OR (95%CI)	*P* value
Total patients	84, 80.8 (71.9–87.8)	20, 19.2 (12.2–28.1)		
Gender and/or sexual orientation				
Female	30, 36.1 (25.9–47.4)	4, 20.0 (5.73–43.7)	**Reference**	
Men reporting female partners only	14, 16.9 (9.5–26.7)	8, 40.0 (19.1–64.0)	4.29 (1.1–16.7)	0.036
Men reporting sex with men (GBMSM)^a^	40, 47.0 (36.6–58.8)	8, 40.0 (19.1–64.0)	1.54 (0.4–5.5)	0.513
Site of infection				
Cervicovaginal	27, 32.1 (22.4–43.2)	3, 15.0 (3.2–37.9)	**Reference**	
Anorectal	9, 9.6 (4.3–18.1)	1, 5.0 (0.0–24.9)	1.00 (0.1–10.9)	1.000
Urethral/first pass urine^b^	48, 58.3 (47.1–69.0)	16, 80.0 (56.3–94.3)	3.00 (0.8–11.2)	0.103
Symptom status				
Asymptomatic	37, 44.0 (33.2–55.3)	9, 45.0 (23.1–68.5)	**Reference**	
Symptomatic	47, 56.0 (44.7–66.8)	11, 55.0 (31.5–76.9)	0.96 (0.4–2.7)	0.939
Failure of previous antimicrobial regimens^c^				
No	12, 14.5 (7.7–23.9)	4, 20.0 (5.7–43.7)	**Reference**	
Yes	71, 85.5 (76.1–92.3)	16, 80.0 (56.3–94.3)	0.68 (0.2–2.4)	0.541
Adherence				
No reported missed doses	79, 94.0 (86.7–98.0)	19, 95.0 (75.1–99.9)	**Reference**	
Missed 1–6 doses	3, 3.6 (0.7–10.1)	1, 5.0 (0.0–24.9)	0.72 (0.1–7.3)	0.783

*P* values calculated using univariable logistic regression.

^a^Includes one transgender male reporting sex with men and women.

^b^One man who had both urine and anorectal infection, only included urine.

^c^One patient with unknown or missing previous treatment regimen.

OR, odds ratio.

Last, we compared cure estimates for the combination regimen to cure reported following minocycline monotherapy from our recent analysis of 123 patients.^5^ The 14-day combination regimen without preceding doxycycline achieved 76.7% cure, but this was not statistically superior to 14 days of minocycline monotherapy (67.5%, *P* = 0.195; Table 4). However, when the combination regimen was preceded by doxycycline, microbial cure was 90.3%, and this was significantly more effective than 14 days of minocycline monotherapy (*P* = 0.013, Table 4).

**Table 4.** Proportion cured versus failed following 14 days of minocycline monotherapy compared to the combination regimen

Antibiotics	Cured *n*, (%)	Failed *n*, (%)	Comparison of all three treatments *P* value	Pair-wise comparisons with minocycline monotherapy *P value*
14 days Minocycline monotherapy^a^	83 (67.5)	40 (32.5)	*P* = 0.025	Reference
14 days minocycline and metronidazole (without doxycycline pre-treatment)	56 (76.7)	17 (23.3)		*P* = 0.195
Doxycycline followed by 14 days of minocycline and metronidazole^b^	28 (90.3)	3 (9.7)		*P* = 0.013

*P* values were calculated using Fisher’s exact test.

^a^Data were obtained from cure following 14 days minocycline for the treatment of *Mycoplasma genitalium* conducted at MSHC between 2020 and 2022 by Clarke *et al.*^5^

^b^Patients received 3–7 days of doxycycline before commencing combination therapy with minocycline and metronidazole.

### Adherence and side effects

Our analysis of microbial cure was restricted to individuals who reported taking at least 50% (i.e. >6 days) of their prescribed regimen, and nine patients who took <50% of the combined regimen were excluded. Among the 104 patients who took >50% of doses, adherence was high: 98 out of 102 (96%) with known adherence data and reported taking all doses, while only 4 out of 102 (4%) missed up to six doses of either drug. Following the combination regimen, nearly half of patients (*n* = 49, 47%) reported no side effects, while 55 (53%) experienced one or more side effects (Table 5). Gastrointestinal side effects included: nausea (*n* = 15, 14%), vomiting (*n* = 5, 5%), diarrhoea (*n* = 11, 11%) and abdominal pain (*n* = 4, 4%). Two patients reported having a metallic taste. Central nervous system (CNS) side effects included headache (*n* = 19, 18%), dizziness (*n* = 18, 17%), fatigue/lethargy/brain fog (*n* = 18, 17%) and mood change (*n* = 7, 7%). Other reported side effects included photosensitivity (*n* = 3, 3%) and insomnia (*n* = 2, 2%). Unfortunately, consistent data grading symptom severity was not available for this study population. Of note, the nine patients who were excluded from analyses because they took <50% of the combination regimen reported discontinuation due to treatment limiting side effects. Almost all these patients reported CNS side effects (details summarized in Figure 1).

**Table 5.** Side effects of combination minocycline and metronidazole

		14 days minocycline and metronidazole (*n* = 104)
Side effects		*n*, % (95%CI)
Gastrointestinal	Nausea	15, 14.4 (8.3–22.7)
	Vomiting	5, 4.8 (1.5–10.9)
	Diarrhoea	11, 10.6 (5.4–18.1)
	Abdominal pain	4, 3.8 (1.1–10.0)
	Reflux/heartburn	0
	Metallic taste	2, 1.9 (0.2–7.8)
CNS	Headache	19, 18.3 (11.4–27.1)
	Dizziness	18, 17.3 (10.6–26.0)
	Fatigue/lethargy/brain fog	18, 17.3 (10.6–26.0)
	Mood changes	7, 6.7 (2.7–13.4)
Other side effects	Insomnia	2, 1.9 (0.2–6.8)
	Photosensitivity	3, 2.9 (0.5–8.2)
	Tendon pain	0
	No side effects	49, 47.1 (37.2–57.2)
	Other	6, 5.8 (2.1–12.1)^a^
	Not available (missing)	3, 2.9 (0.5–8.2)

^a^Rash to scrotum, palpitation, unsteady gait, dry mouth, visual field changes, myalgia.

## Discussion

This prospective analysis, which captured all patients attending a large sexual health service with a macrolide-resistant *M. genitalium* infection who received a combination of 14 days of minocycline and metronidazole, demonstrated microbial cure in 80.8% (95% CI: 71.9–87.8%) of patients. Forty-six percent of patients had failed two or more previous regimens, representing a heavily pre-treated population. The overall proportion of patients cured using the combination regimen appeared to be influenced by preceding doxycycline therapy, which reflects real-world clinical practice, as it is commonly used syndromically and in resistance-guided therapy for *M. genitalium.*^13,14^ Although limited by small numbers, our data suggest that the combination regimen could be more effective than minocycline monotherapy, and that efficacy is likely to be further enhanced by pre-treatment with doxycycline. In our previous study of 123 patients who received minocycline monotherapy for 14 days, 67.5% (95% CI: 58.4–75.6%) experienced microbial cure.^5^ In our current cohort, among 73 patients treated with 14 days of minocycline combined with metronidazole, 76.7% (95% CI: 65.4–85.8%) experienced cure. Interestingly, the addition of up to 7 days of doxycycline preceding the 14 days of combination minocycline and metronidazole among 31 patients achieved microbial cure in 90.3% (95% CI: 74.2–98.0%), which was significantly more effective than minocycline monotherapy for 14 days. Larger studies are clearly needed to determine whether doxycycline, which has been shown to lower bacterial load before commencement of a curative antibiotic,^13^ does significantly improve cure above 14 days of the combination regimen alone. *M. genitalium* is a slow growing organism and it is biologically plausible that longer duration regimens may be needed to eradicate infection and achieve cure.

The use of metronidazole for *M. genitalium* is supported by recent *in vitro* data demonstrating the susceptibility of *M. genitalium* to metronidazole [mean inhibitory concentrations (MICs) ranging from 1.6–12.5 mg/L], tinidazole (MICs 0.8–6.3 mg/L) and secnidazole (MICs 3.1–12.5 mg/L).^11^ This line of investigation followed findings from a study of acute pelvic inflammatory disease where cervical *M. genitalium* was detected in 4% of patients treated with metronidazole, doxycycline and ceftriazone compared to 14% of patients treated with only doxycycline and ceftriazone.^10^ The overall proportion of *M. genitalium* infections cured with combined minocycline and metronidazole compares favourably to pristinamycin (75%, 95% CI: 66–82%) a drug with very high cost and limited availability,^7^ although the latter has the distinct advantage of being able to be used in pregnancy and while breastfeeding. In our population where the prevalence of the S83I mutation is approaching 25%,^15^ cure rates from this combination regimen are currently similar to the proportion cured with sitafloxacin (80.6%, 95% CI: 74.9–85.5%) but as with pristinamycin, sitafloxacin is expensive with limited availability.^6^ In this study, men reporting only female partners had higher odds of treatment failure compared to women. Further studies are needed to determine whether this was due to the nature of the sample, anatomical site, host factors or other unmeasured behavioural factors.

Both minocycline and metronidazole are widely used in the community for various infections, are easily accessible, and their safety profiles are well established. Commonly reported side effects of minocycline include abdominal cramps, vestibular toxicity (such as weakness, dizziness, vertigo and ataxia) and headaches.^16^ Metronidazole is primarily associated with gastrointestinal symptoms and headaches.^16^ To assist clinicians in understanding the relative frequency of side effects reported with this combination regimen, we also tabulated the side effects reported by patients receiving minocycline monotherapy twice daily for 14 days for *M. genitalium* infection in our previous study,^5^ and by male and female participants who received oral metronidazole twice daily for 7 days in partner treatment trials for bacterial vaginosis (Supplementary Table S1, available as Supplementary data at *JAC* Online).^17,18^ Overall, we found that the combination of minocycline and metronidazole was associated with a higher proportion of patients reporting CNS and gastrointestinal side effects compared to those taking either drug alone (Table 5 and Supplementary Table S1). Nausea (14%) and diarrhoea (11%) appeared to be more frequently reported with the combination regimen than with minocycline monotherapy (6% and 2%, respectively). Additionally, headaches, dizziness and fatigue were more commonly reported with the combination regimen (17%–18%) compared to metronidazole (1%–13%) or minocycline (4%–8%) monotherapy. Interestingly, a similar proportion of patients (45%–63%) reported no side effects with either the combination regimen or monotherapy with either drug. Limitations of this comparison include the absence of data to grade symptom severity and that the regimens were not directly compared to each other within the same study, with potential differences between patient populations impacting on findings.

Overall, the combination regimen demonstrated similar efficacy to sitafloxacin, and possibly superior efficacy compared to minocycline alone, suggesting it may have a role as an affordable quinolone-sparing option for patients who do not experience or only experience mild side effects. Data on the efficacy and tolerability of combining metronidazole with minocycline offers clinicians valuable information at a time when therapeutic options are extremely limited and contracting.

A strength of this study is that it provides the first data on combining metronidazole with minocycline for the treatment of *M. genitalium* infections. However, our sample size was relatively small and represented a heterogenous group of cases, which limited our ability to accurately identify factors associated with treatment failure and determine whether pre-treatment with doxycycline was statistically superior to the combination alone. Additionally, this prospective analysis relied on patients’ self-reported symptoms, adherence and risk of reinfection, which may introduce recall bias. While we collated side effect data and were able to compare it to either of the drugs alone, data on severity of side effects were not available. In line with our previous studies,^5,6^ 15% of patients were lost to follow up. As patients were not participating in a randomized trial, no direct comparison with other treatment options was able to be conducted and all comparisons are with previous collected data. Finally, half of the patients in our study were heavily pre-treated (i.e. had received two or more previous regimens) and therefore, the results may not be generalizable to a general population.

In summary, the combination of minocycline and metronidazole for 14 days cured ∼80% of macrolide-resistant *M. genitalium* infections. Our data suggest that preceding doxycycline could further improve cure, although larger studies are needed. The primary advantage of the combined minocycline and metronidazole therapy is that both drugs are relatively affordable and widely available. However, this regimen is associated with a notable increase in CNS and gastrointestinal side effects, and therefore may be best restricted to cases where no alternative drugs are available, or quinolones are contraindicated. Clinicians should discuss potential side effects with their patients and ensure they are aware of the need to discontinue therapy and contact their doctor if they arise.

## Supplementary Material

dkaf142_Supplementary_Data

## Supplementary data

Supplementary Table S1 is available as Supplementary data at *JAC* Online.
