# Supplementary material for: Efficacy and tolerability of the combination of minocycline and metronidazole for macrolide-resistant Mycoplasma genitalium
Source: J Antimicrob Chemother. 2025 May 22;80(7):1878–84. doi: 10.1093/jac/dkaf142 (PMC12209785; doi:10.1093/jac/dkaf142)
Supplement: dkaf142_Supplementary_Data [file dkaf142_supplementary_data.docx]

**Supplementary Table 1. Side effects of 7days metronidazole monotherapy for treatment of bacterial vaginosis and 14 days minocycline monotherapy for Mycoplasma genitalium infection**

|  |  | **7 days Metronidazole**  **(N=96)^a^** | **14 days Minocycline monotherapy (N=90)^b^** |
| --- | --- | --- | --- |
|  |  | **n, % (95%CI)** | **n, % (95%CI)** |
| **Gastrointestinal** | Nausea | 17, 17.7 (10.7- 26.8) | 5, 5.6 (1.7-11.9) |
|  | Vomiting | 0 | 0 |
|  | Diarrhoea | 0 | 2, 2.1 (0.2-7.4) |
|  | Abdominal pain | 1, 1.0 (0.02- 5.7) | 3, 3.2 (07-9.0) |
|  | Reflux/heartburn | 0 | 2, 2.1 (0.2-7.4) |
|  | Metallic taste | 15, 15.6 (9.0-24.5) | 0 |
| **Central Nervous System** | Headache | 12, 12.5 (6.6-20.8) | 5, 5.6 (1.7-11.9) |
|  | Dizziness | 1, 1.0 (0.02- 5.7) | 8, 8.4 (3.7-15.9) |
|  | Fatigue/lethargy/brain fog | 6, 6.3 (2.3- 13.1) | 4, 4.2 (1.2-10.4) |
|  | Mood changes | 0 | 3, 3.2 (07-9.0) |
| **Other side effects** | Insomnia | 0 | 0 |
|  | Photosensitivity | 0 | 1, 1.1 (0.02-5.7) |
|  | Tendon pain | 0 | 0 |
|  | No side effects | 60, 62.5 (52.0-72.2) | 43, 45.3 (35.0-55.8) |
|  | Other | 22, 22.9 (15.0-32.6)^c^ | 7, 7.4 (3.0-14.6)^d^ |
|  | Not available (missing) | 0 | 15, 15.8 (9.1-24.7) |

^a^ Data for side effects of 7 days metronidazole monotherapy was obtained from men and women in male partner treatment trials for bacterial vaginosis between 2015 to 2019 at Melbourne Sexual Health Centre, Plummer *et al.* ^17,18^

^b^ Data for side effects of 14 days minocycline was obtained from a study of *Mycoplasma genitalium* between 2020 and 2022 at Melbourne Sexual Health Centre by Clarke *et al*.^5^

^c^ Vaginal dryness, vaginal irritation, thrush, increased appetite, tingling sensation in hands, tension behind eyes and feeling ill, stomach upset, mild generalised body rash

^d^ Rash, bloating, sleep disturbance, dry eyes, derealisation
